# Influence of Association Network Properties and Ecological Assembly of the Foliar Fugal Community on Crop Quality

**DOI:** 10.3389/fmicb.2022.783923

**Published:** 2022-04-05

**Authors:** Lei Xing, Qiqi Zhi, Xi Hu, Lulu Liu, Heng Xu, Ting Zhou, Huaqun Yin, Zhenxie Yi, Juan Li

**Affiliations:** ^1^College of Agronomy, Hunan Agricultural University, Changsha, China; ^2^Great Wall Cigar Factory, China Tobacco Sichuan Industrial Co., Ltd, Shifang, China; ^3^School of Minerals Processing and Bioengineering, Central South University, Changsha, China

**Keywords:** microbial community, phyllosphere, fungi, network, phylogenetic structure, null model, crop quality

## Abstract

Revealing community assembly and their impacts on ecosystem service is a core issue in microbial ecology. However, what ecological factors play dominant roles in phyllosphere fungal community assembly and how they link to crop quality are largely unknown. Here, we applied internal transcriptional spacer high-throughput sequencing to investigate foliar fungal community assembly across three cultivars of a Solanaceae crop (tobacco) and two planting regions with different climatic conditions. Network analyses were used to reveal the pattern in foliar fungal co-occurrence, and phylogenetic null model analysis was used to elucidate the ecological assembly of foliar fungal communities. We found that the sensory quality of crop leaves and the composition of foliar fungal community varied significantly across planting regions and cultivars. In Guangcun (GC), a region with relatively high humidity and low precipitation, there was a higher diversity and more unique fungal species than the region of Wuzhishan (WZS). Further, we found that the association network of foliar fungal communities in GC was more complex than that in WZS, and the network properties were closely related to the sensory quality of crop. Finally, the results of the phylogenetic analyses show that the stochastic processes played important roles in the foliar fungal community assembly, and their relative importance was significantly correlated with the sensory quality of crop leaves, which implies that ecological assembly processes could affect crop quality. Taken together, our results highlight that climatic conditions, and plant cultivars play key roles in the assembly of foliar fungal communities and crop quality, which enhances our understanding of the connections between the phyllosphere microbiome and ecosystem services, especially in agricultural production.

## Introduction

Assembly patterns within microbial communities are an important topic in microbial ecology and are closely related to the functioning of plant-associated ecosystems (Konopka, 2009). There are many different factors that could affect the structure of the plant-associated microbial community. For example, soil microbial communities were different across various plant species (Ma et al., 2019), because of the range of nutrient content available in the leaf and root litter that alters decomposer abundance (Otsing et al., 2018). The foliar endophytic fungal community in *Cirsium arvense* could be associated with the soil nutrients and arbuscular mycorrhizal (AM) colonization (Eschen et al., 2010), and the latter would be further affected by root exudates, such as methyl salicylic acid and acibenzolar-*S*-methyl (Mannaa et al., 2020). Furthermore, climate change, such as warming, would decrease fungal species richness and change foliar fungal community composition, especially at the end of the growing season (Faticov et al., 2021). In the case of elevated atmospheric carbon dioxide, the growth of trees would also lead to the changes in the composition of microbial communities that colonize the fallen leaves (Kelly et al., 2010). Crop cultivar, tissue type, and climatic factors can all significantly influence phyllosphere fungal community structure; moreover, location-dependent climate conditions could contribute to the differences in abundance, diversity, and presence of genera containing pathogens, whereas the root communities were less affected by climatic factors (Latz et al., 2021). In addition, drought changes the composition of the root microbiome, where changes in the relative abundance of specific bacterial groups were associated with increased drought tolerance in plants (Fitzpatrick et al., 2018). Although bacterial abundance was negatively affected by O_3_ stress, it was found that the fungal abundance was substantially stimulated (up to 12-fold compared with non-fumigated plants at 20°C). These changes were accompanied by modifications of the genetic structures and a relative increase in amino acids catabolism (Changey et al., 2018). The above findings advanced our understanding of the drivers in shaping plant-associated communities, but the mechanisms of assembly in the foliar fungal community remain largely unknown.

Exploring network assembly in microbial communities and their responses to environmental changes is fundamentally vital for the understanding of community organization (Zhou et al., 2010). Microbial community networks can provide a mechanistic association between species in a specific environment and information on the dynamics of community structure as a function of time or other external variables (Cardona et al., 2016). For example, climate change, such as warming, can significantly increase network complexity, including network size, connectivity, and number of keystone species (Faticov et al., 2021), whereas elevated CO_2_ can increase modularity and hierarchy (Zhou et al., 2010). The community assembly of plant-associated microbes may have some differences, such as the rhizosphere microbial networks. In the rhizosphere, complexity of microbial ecological network increases with the growth of wild oat plants (Shi et al., 2016). *Artemisia annua* (sweet wormwood) promoted a specific root-associated microbial community assembly process, with increased abundance of plant growth–promoting microorganisms and building of interkingdom association networks, which may be beneficial for the fitness of plants in the natural environment (Shi et al., 2021). Together, these results revealed that network assembly of plant-associated microbial communities could be closely related to plant growth. However, how network assembly of the phyllosphere microbial community is affected by climatic conditions and crop cultivars have been less well studied.

Phylogenetic analyses based on null model provide a conceptual background for understanding the ecological processes of community assembly that determine which, and how many, species live in a particular environment (Campbell et al., 2011). Foliar fungi are of great importance to host plant growth and health and can also affect ecosystem functions. Most importantly, host environmental filtering caused by fungal infections outweighs competitive exclusion in driving foliar fungal community assembly in symptomatic leaves (Liu X. et al., 2021). Community co-occurrence theories can be explained mainly by niche-based theory and the null model (Gravel et al., 2006; Jiao et al., 2020). On the one hand, niche-based theory (Zhou and Ning, 2017) posits that deterministic processes play a key role in the community assembly process. Different species occupy different niches, and ecological selection can affect the community co-occurrence. On the other hand, the neutral model demonstrates that all species are equivalent on ecological function, and the community assembly is affected by stochastic processes but not their ecological abilities. The environment can play a vital role in stochastic processes that correlate with community assembly. However, the deterministic and stochastic processes that shape phyllosphere fungal community assembly have not been extensively explored.

The present study aims to reveal the assembly of phyllosphere fungal communities inhabiting a Solanaceae (tobacco) crop across climatic conditions and cultivars. We set up a large-scale field experiment with three crop cultivars and in areas with different climatic conditions in Hainan, China. We assessed the sensory quality of crop leaves and explored the phyllosphere fungal communities using internal transcriptional spacer (ITS) high-throughput sequencing technology. We hypothesized that (i) the sensory quality of crop leaves and the composition of the foliar fungal community are significantly affected by both the planting region and cultivar; (ii) the association network of foliar fungal communities and its characteristics are closely related to the sensory quality of the crop; (iii) the community assembly of all samples would be dominated by the ecological drift, which is community phylogeny structure with little effect on the sensory quality of the crop.

## Materials and Methods

### Experimental Description

Three cultivars of the Solanaceae crop tobacco, including Haiyan 101 (HY101), Haiyan 201 (HY201), and Haiyan 109 (HY109), were used to conduct a field experiment in two regions of Hainan, China, at Guangcun (GC) town, Danzhou (19°49′ N, 109°28′ E) and Panyang town, Wuzhishan (WZS) City (18°87′ N, 109°40′ E). The three selected cultivars are core cultivars with consistent disease resistance and agronomic characteristics; moreover, each cultivar has different sensory quality traits. GC town is located in the northwest of Hainan, at an elevation of 51.6 m; it is a semihumid region. Mountain of five fingers (WZS), with an elevation of 154 m, is located on the central line of Hainan, which is the highest point in Hainan Province and is a humid mountain region. During the crop growth period in GC town, the average temperature was 23°C, average humidity was 84%, total precipitation was 89 mm, and the average photosynthetically effective radiation was 306 μmol m^–2^ s^–1^. In WZS, the average temperature was 23°C, average humidity was 78%, total precipitation was 132 mm, and the average photosynthetic effective radiation was 338 μmol m^–2^ s^–1^.

The experiment was conducted from November 2018 to December 2019. The plants were transplanted in GC town on January 20 and in Panyang town on January 13. The experiment adopted a random block design with three replicates, the plot area was 90 m^2^, and the row spacing was 40 × 100 cm. Other field management measures were carried out in accordance with local planting practices (Lei et al., 2021).

On April 17, 2019, the middle part of the fresh blade was collected at the maturity stage of the crop. A total of 18 plants were randomly selected from each plot, and the middle leaves of every sixth plant were taken as a sample, and each sample was divided into two parts. One part was kept at 4°C and brought back to the laboratory for subsequent indoor foliar microbial DNA extraction experiments. The other part was used to evaluate the sensory quality of the leaves.

### Sensory Qualities of Crop Leaves

The evaluation in sensory quality of leaf aroma substances was based on the Sichuan China Tobacco “mellow and sweet” category to construct a leaf raw material evaluation table (9-point system), from which the average level was taken. The main evaluation contents were as follows: (i) coordination of flammability indicators: a sense of balance; (ii) combustion characteristics: gray, condensed gray, and combustibility; (iii) the mellowness of smoke: lingering, fullness, and smoothness; (iv) the mellowness of aroma: maturity, richness, and mellowness; (v) the mellow aftertaste: irritation, aftertaste, sweetness, and cleanliness; (vi) miscellaneous gas: woody gas, soil fishy gas, green mixed gas, burnt gas, and protein smell; (vii) fragrance: hay, floral, cellar, milk, woody, bean, sweet, honey, leather, baking, normal, resin, powder, and burnt sweet.

### DNA Extraction and High-Throughput Sequencing

Fifteen grams of leaf samples obtained from various parts of the leaf surface (avoiding the main and branch veins) using a sterile puncher was added to 50 mL of 0.1% Tween-80 bacterial phosphate buffer (pH 7.0). The samples were then shaken for 30 min at 170 revolutions/min (rpm) and 28°C, the bacterial suspension was collected, and the leaf samples were washed twice more. The collected suspensions were centrifuged for 15 min (4°C, 10,000 rpm) to pellet the microorganisms. The pellet was suspended in sterile water and washed three times. Finally, the microorganisms were resuspended with 1 mL of sterile water for subsequent DNA extraction. After the above treatments, the leaves were rinsed three times with sterile water, with the last rinsing solution (1 mL) spread on LB plate medium and cultured in an incubator at 30°C for 2 days to determine whether the microorganisms on the surface of the leaves were completely eluted.

Genomic DNA extraction of foliar microorganisms was performed using the Plant Genomic DNA Kit (Plant Genomic DNA Kit) following the manufacturer’s protocol. We used the primer pair ITS1-1F-F/ITS1-1F-R with barcodes to amplify the ITS. Amplicons were sequenced by Illumina NovaSeq platform with a Plant Genomic DNA Kit (2 × 250–base-pairs [bp] paired ends). The raw sequencing data were deposited in the NCBI Sequence Read Archive database according to accession number PRJNA778452.

### Sequencing Processing and Statistical Analyses

Raw sequences were split into sample libraries with perfect matches to barcodes. Low-quality sequences with QC < 20 over a 5-bp window size were trimmed using Btrim, and sequences with a length of < 100 bp were removed (Kong, 2011). Then, the forward and reverse sequences were spliced together. Any sequences containing ambiguous bases or the incorrect length were removed, and remaining sequences were compared against the UNITE v8.2 database (Kõljalg et al., 2005) to remove possible chimeras. The length of the sequencing fragment was 200–400 bp. Then, UPARSE (Edgar, 2013) was used to cluster and produce operational taxonomic units (OTUs) at 97% similarity level. In order to ensure the authenticity of the data, we removed OTUs that were represented by only one sequence in overall data (global singletons). For comparability between different samples in subsequent data analysis, the ITS sequences were resampled to 10,000 per sample. Finally, RDP Classifier (Wang et al., 2007) was used to perform online comparison and systematic taxonomic annotation of the ITS sequences. The above analyses were conducted on a Galaxy Illumina sequencing analysis platform (Zhou et al., 2016) publicly available at http://zhoulab5.rccc.ou.edu:8080/.

### Network Construction

To construct a microbial association network, correlations between pairwise OTUs that were present in more than a half of the samples were calculated using the SparCC method (Friedman and Alm, 2012; Preheim et al., 2013; Ju and Zhang, 2015; Watts et al., 2019) using the “microeco” v0.3.1 package (Liu C. et al., 2021) in R. Only significant correlations (*p* < 0.01) larger than 0.3 were retained for network construction. Network analyses were conducted in “igraph” v1.2.6 package (Ju et al., 2016). Some traits of association networks such as nodes, links, density, transitivity, modularity, centralization of degree, the average path distances, and diameter based on these methods were analyzed. The impact of ecological environment factors and crop cultivar on the structure of the foliar fungal community using permutational multivariate analysis of variance (PERMANOVA) using distance matrices (Alekseyenko, 2016).

### Phylogenetic Analyses Based on Null Model

The fungal phylogenetic tree was constructed by FastTree v2.1.11 (Price et al., 2010). Considering the high degree of variation of ITS sequences, we performed constrained topology search based on a guide tree, as reported previously (Nuccio et al., 2016). The guide tree was built from the full-length SSU sequences of 386 representative species with one representative for each fungal family. The SSU sequences were from Silva 138.1 Ref NR database (Quast et al., 2013). The nearest taxon index (NTI, i.e., negative of the standardized effect sizes of mean nearest taxon distance) and net relatedness index (NRI, i.e., negative of the standardized effect sizes of mean pairwise phylogenetic distance) of foliar fungal communities were calculated using “picante” v1.8.2 package in R (Kembel et al., 2010). The phylogenetic signals between closely species were estimated by Mantel correlogram (Borcard and Legendre, 2012). The ecological assembly process of the microbial community was assessed by using “microeco” v0.3.1 package (Stegen et al., 2013, 2015; Liu C. et al., 2021). The visualization of molecular ecological network was realized by Gephi 0.9.1 (Hernandez-Garcia et al., 2016).

### Statistical Analysis

All packages discussed here for analyses were run in R v3.6 environment (Ihaka and Gentleman, 1996; R CoreTeam, 2013). The α diversity indices, including Shannon, Simpson, inverse Simpson, and Chao1, were calculated by using “diversity” function in the “vegan” v2.5-6 package (Dixon, 2003). The effects of crop cultivars and planting regions on microbial community structure were analyzed by principal components analysis (PCA) based on weighted UniFrac distance using the “rda” function. Samples were clustered based on sensory quality by using the “heatmap” function in R (Zhao et al., 2014). Venn plot analyses were conducted by using “VennDiagram” v1.6.20 (Chen and Boutros, 2011). For the correlations between sensory quality and diversity, phylogenetic indices were calculated by Pearson correlation. The differences in diversity and network properties were tested by least significant difference test.

## Results

### Sensory Quality of Crop Leaves

The sensory quality of crop leaves was significantly affected by cultivar and ecological region. We estimated sensory quality indices using seven aspects, including coordination of flammability indicators, combustion characteristics, mellowness of smoke, mellowness of aroma, mellow aftertaste, miscellaneous gas, and fragrance. Our results showed that the combustibility of HY201 and HY109 displayed good performance in both planting regions, whereas HY101 had the lowest scores. Fragrance was a key indicator that determined the style and characteristics of crop leaves. On the whole, the samples from GC mainly displayed a sweet fragrance, whereas hay fragrance was more prominent in the samples from WZS. GC-HY201 had the highest scores in the mellowness of aroma, the mellowness, and the purity of aftertaste, and its corresponding sense of balance was also the highest, which showed obvious differences with WZS-HY201 and indicated that ecological environment had a great influence on the aroma of HY201.

**FIGURE 1:**
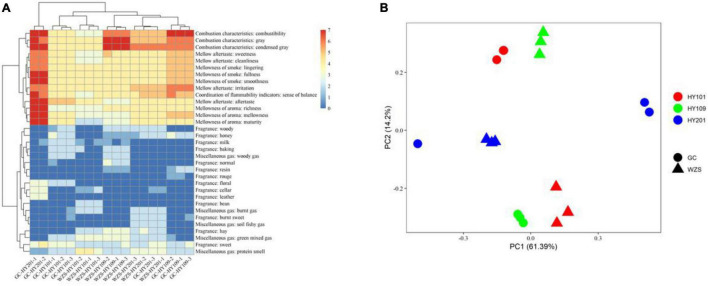
**(A)** Heatmap for sensory quality of crop leaves. Color represents the scores of sensory qualities. Clustering analysis was based on Euclidean distance. **(B)** Principal components analysis for sensory quality of crop leaves across three crop cultivars and two planting regions. Color denotes different crop cultivars. Shape denotes different regions.

The multiplicity in sensory quality across planting regions was differed with cultivar. The results of clustering analysis based on euclidean distance showed that the quality of WZS-HY201 was close to that of WZS-HY109, whereas GC-HY101 was close to that of WZS-HY101, which indicated that these two pairs of samples had, respectively, similar sensory quality (Figure 1A). The PCA results of sensory quality scores suggested that the sensory quality varied significantly across planting regions and cultivars, and the impact of planting regions was greater than that of cultivars (Figure 1B).

### Associations Between Fungal Diversity and the Sensory Quality on Crop Leaf Surface

**FIGURE 2:**
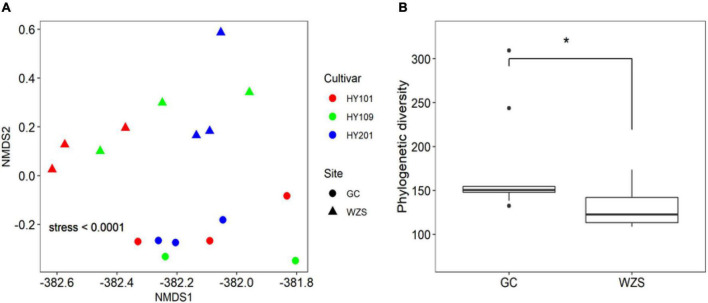
**(A)** Non-metric multidimensional scaling (NMDS) based on Bray–Curtis similarity index of foliar fungal community. The composition of foliar fungal community varied significantly across planting regions and cultivars. **(B)** Difference of fungal phylogenetic diversity based on Kruskal–Wallis test between the two regions (GC and WZS, *p < 0.05).

The results of non-metric multidimensional scaling of foliar fungal community showed that the composition of foliar fungal community varied significantly across planting regions and cultivars (Figure 2A). GC, with relative high humidity and low precipitation, had higher diversity, but more unique fungal species, than the region of WZS. The boxplots with stars showed the correlation between the phylogenetic diversity (PD) and the two regions (Figure 2B). As for the association between fungal diversity and sensory quality, the heatmap results indicated that the observed species number and Chao1 were significantly correlated (*p* < 0.01) with the mellowness of aftertaste, fragrance, and smoke, such as the cleanliness, sweetness, and mellowness (Supplementary Figure 1). Simpson diversity was significantly correlated (*p* < 0.01) with the hay fragrance, and inverse Simpson diversity was significantly correlated (*p* < 0.01) with maturity, aftertaste, and mellowness (Supplementary Figure 1). Linear regression analyzed the relationships between PD and differences in sensory quality of crop leaves (Supplementary Figure 2; the solid lines represent *p* < 0.05). In terms of the overall fragrance certain honey aroma, floral, cellar, and leather fragrances were observed in GC HY201, whereas hay and woody fragrances were more prominent among the samples of WZS HY201. In summary, the diversity of leaf fungi had a close relationship to its sensory quality.

### Microbial Co-occurrence of Fungi on Leaf Surface

**FIGURE 3:**
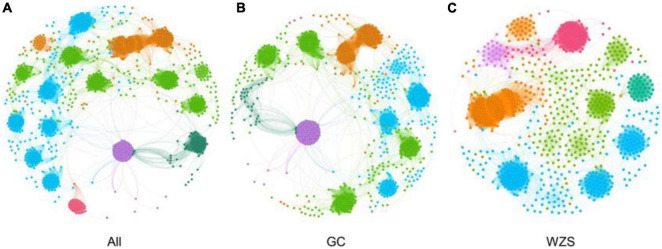
The association network of foliar fungal community. Node color represents different modules. The network pattern was visualized by Gephi software. **(A)** The network of all samples in two regions. **(B)** The subnetwork of the samples in GC. **(C)** The subnetwork of the samples in WZS.

**FIGURE 4:**
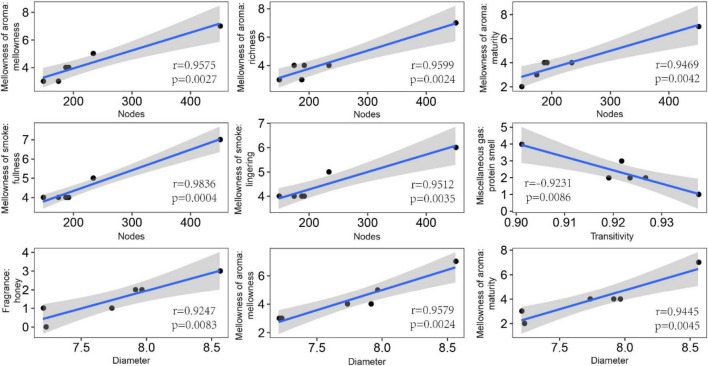
Pearson correlations between the network properties and the sensory quality of crop leaves. The shadow indicates the 95% confidence interval. The miscellaneous gas: protein smell was significantly related with the transitivity (*p* < 0.01). The fragrance: honey, the mellowness of aroma: mellowness, and maturity were significantly related with diameter (*p* < 0.01).

In order to explore the leaf fungal association networks, we applied the SparCC method (Watts et al., 2019) to construct molecular ecology networks of foliar fungi across all samples (Figure 3). Further the trendline with scatter plots suggested the correlation between the network properties and the sensory quality of crop (Figure 4). The mellowness of aroma: mellowness, richness and maturity, and the mellowness of smoke: fullness and lingering were significantly related with the nodes (*p* < 0.01). The miscellaneous gas: protein smell was significantly related with the transitivity (*p* < 0.01). The fragrance: honey and the mellowness of aroma: mellowness and maturity were significantly related with diameter (*p* < 0.01). A subnetwork for each sample was extracted based on the presence of nodes, and the associations between their properties and sensory quality were accessed by a linear model (Table 1). Our results showed that the number of nodes and connections of ecological networks in GC was higher than those in WZS. The subnetwork of GC-HY109 had the greatest number of nodes (450), followed by GC-HY201 (233), WZS-HY109 (191), GC-HY101 (186), WZS-HY201 (173), and WZS-HY101 (148). In GC, the number of connections for HY101, HY109, and HY201 were 2,705, 90,087, and 3,076, respectively. While for WZS, the numbers of connections for HY101, HY109, and HY201 were 1,272, 3,517, and 2,739, respectively. The percentage of positive associations among treatments had no significant differences, whereas the modularity values of WZS-HY101 and GC-HY201 were 0.312 and 0.359, respectively, which were larger than those of GC-HY101 (0.124), GC-HY109 (0.17), WZS-HY109 (0.257), and WZS-HY201 (0.151). These results indicated that the size of subnetworks in GC was larger and more complex than those in WZS and that climatic conditions could affect the network characteristics. The average path length of HY201 was 3.518, followed by HY101 (2.948), and HY109 (2.591) in the GC planting region, which showed that the response speed of GC-HY201 leaf fungi to the external environment was lower than GC-HY101 and GC-HY109 and that the plant cultivar can affect the foliar fungal subnetwork structure of crop leaves. Furthermore, we found that 99.67% of the network nodes (2,093) were peripheral nodes, with only 0.33% of nodes (7) being module hubs, and no network hubs and connectors were detected. Module hubs in the fungal networks included *Ilyonectria destructans* (OTU 298), *Penicillium brunneoconidiatum* (OTU 566), *Kodamaea ohmeri* (OTU 1026), *Solicoccozyma terricola* (OTU 1898), and *Penicillium maclennaniae* (OTU 1989).

**TABLE 1:** Topological properties of molecular ecological subnetworks in foliar fungal communities on crop leaves across crop cultivars and plating regions.

Group	Nodes	Links	Density	Transitivity	Modularity	Centralization of degree	Average path distances	Diameter
GC-HY101	186 (18)^a^	2,705 (585)^a^	0.156 (0.015)^a^	0.919 (0.024)^a^	0.124 (0.02)^a^	0.242 (0.004)^a^	2.948 (0.248)^a^	7.911 (0.937)^a^
GC-HY109	450 (317)^a^	90,087 (145,523)^a^	0.367 (0.366)^a^	0.937 (0.057)^a^	0.17 (0.215)^a^	0.208 (0.092)^a^	2.591 (1.2)^a^	8.561 (2.976)^a^
GC-HY201	233 (24)^a^	3,076 (1,294)^a^	0.113 (0.046)^a^	0.923 (0.047)^a^	0.359 (0.191)^a^	0.179 (0.047)^a^	3.518 (0.64)^a^	7.96 (1.251)^a^
WZS-HY101	148 (22)^a^	1,272 (509)^a^	0.125 (0.075)^a^	0.901 (0.021)^a^	0.312 (0.199)^a^	0.195 (0.05)^a^	2.71 (0.569)^a^	7.226 (1.793)^a^
WZS-HY109	191 (99)^a^	3,517 (4,727)^a^	0.123 (0.062)^a^	0.922 (0.064)^a^	0.257 (0.038)^a^	0.215 (0.049)^a^	2.92 (0.517)^a^	7.73 (1.821)^a^
WZS-HY201	173 (14)^a^	2,739 (1,155)^a^	0.178 (0.055)^a^	0.927 (0.026)^a^	0.151 (0.016)^a^	0.246 (0.022)^a^	2.745 (0.337)^a^	7.206 (1.586)^a^

*Values in parentheses are standard deviation (n = 3). The letter label means there are no statistical significances (p < 0.05) of network properties among groups, which is tested by multiple comparisons by means of least significant difference, and p-value is adjusted by the Bonferroni method.*

Further, the network properties were significantly related to the sensory quality of the leaves. For example, we found that the number of nodes was significantly (*p* < 0.05) positively correlated with balance, mellowness of smoke, mellowness of aroma, and leather. The link number had a significantly positive correlation (*p* < 0.05) with fluency, richness, leather, and cellar. Density was positively correlated only with the leather (*p* < 0.05; Supplementary Figure 3). Furthermore, network transitivity had a significantly negative correlation with protein smell, whereas network diameter had a significantly positive correlation with maturity, alcohol, and sweetness (*p* < 0.05; Supplementary Figure 3). Overall, these results indicated that the properties of the leaf fungal microbial ecology networks can affect the leaf sensory quality to some extent.

### Ecological Assembly Processes of the Fungal Community on Leaf Surfaces

**FIGURE 5:**
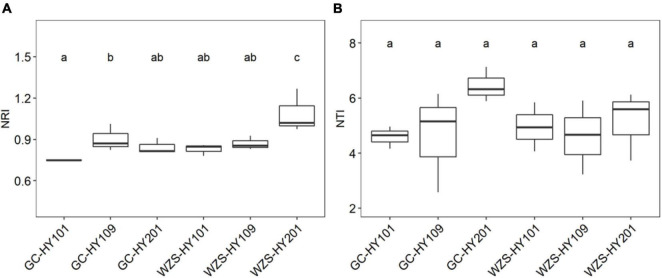
**(A)** Boxplots for net relatedness index (NRI) for foliar fungal communities. **(B)** Boxplots for nearest taxon index (NTI) for foliar fungal communities. Different small letters indicate significant differences (*p* < 0.05).

In order to explore community assembly of the foliar fungal community, we calculated a set of indices based on null model, including NRI, NTI, βNTI, and βNRI (Figure 5 and Supplementary Figure 5; Stegen et al., 2012). We first detected the significant correlations between environmental distance and phylogenetic distances between phylogenetically close species, indicating phylogenetic signal in environment conditions (Supplementary Figure 4). Our results showed that NTI and NRI significantly varied among cultivars and planting regions (Figure 5). For example, the community NRI of fungi on leaf surfaces in GC region was between 0.74 and 1.01, whereas in WZS, it was between 0.78 and 1.26. The NRI values of the WZS fungal communities were larger than those of GC. The community NTI of fungi on leaf surfaces in GC was between 2.58 and 7.13, whereas in WZS, it was between 3.23 and 6.12. This meant that the community assembly of the foliar fungal community was affected by local α diversity. Pearson correlation analysis showed that there were significant relationships between NRI, NTI, PD, and sensory quality (Supplementary Table 1). The results showed that NRI was significantly related to the burnt sweet (*r* = 0.525, *p* < 0.05), the fragrance of milk (*r* = –0.541, *p* < 0.05), and the soil fishy gas (*r* = 0.597, *p* < 0.05); NTI was significantly correlated with the cellar fragrance (*r* = 0.508, *p* < 0.05), whereas the PD was significantly correlated with resin fragrance (*r* = 0.663, *p* < 0.05), rouge fragrance (*r* = 0.907, *p* < 0.05), burnt gas (*r* = –0.500, *p* < 0.05), and combustibility (*r* = 0.514, *p* < 0.05). This result showed that among the sensory quality of the crop, only the fragrance of milk and the soil fishy gas were greatly affected by NRI of the fungus on the leaf surface, and only the cellar fragrance was greatly affected by NTI of the fungus on the leaf surface, whereas the resin fragrance, rouge fragrance, burnt gas, and combustibility were greatly affected by PD of the fungus on the leaf surface. Furthermore, we found that the βNTIs of all foliar fungal communities were larger than 1 and less than 2, whereas the βNRIs of all foliar fungal communities were larger than 0.9 and less than 1.4, and the βNRIs of HY101 and HY109 were significantly different (*p* < 0.05), which indicated that spatial turnover was affected by the stochastic process of foliar fungal community assembly (Supplementary Figure 5; Stegen et al., 2012).

The assembly process of the foliar fungal community in GC-HY201 included drift (67%) and dispersal limitation (33%); moreover, in WZS-HY101 and WZS-HY109, it included drift (67%), limiting dispersal (33%, Table 2). This implied that stochastic processes could markedly influence crop fungal community assembly. The linear regression analysis showed that βNTI was linearly related to the sensory quality of crop leaves, indicating that the foliar fungal community βNTI had correlation with the differences in foliar sensory quality between pairwise samples (Supplementary Figure 5, the solid line represents *p* < 0.05).

**TABLE 2:** Ecological processes of community assembly for foliar fungi of crop leaves.

Sample	Dispersal limitation %	Even dispersal %	Drift %
GC-HY101	0	0	100
GC-HY201	33	0	67
GC-HY109	33	67	0
WZS-HY101	33	0	67
WZS-HY201	67	0	33
WZS-HY109	33	0	67

## Discussion

In our study, we studied various plant cultivars and ecological regions with different climatic conditions in Hainan, China. Our results showed that the sensory quality and foliar fungal community structure showed significant differences across different planting regions and crop cultivars. There were significant associations between the sensory quality of crop leaves and foliar fungal diversity. Furthermore, ecological association networks of crop foliar fungi in GC were more complex than those in WZS, and the community assembly was dominated by ecological drift. Network assembly had significant correlations with sensory qualities, whereas ecological processes of community assembly had little effect on sensory qualities. These results highlighted how the phyllosphere fungal community is closely associated with crop quality.

### Effects of Foliar Fungal Community on Crop Quality

Our results demonstrated that planting regions with different climate conditions and cultivars had significant effects on the sensory quality of a Solanaceae crop, which is likely to be led by the changes in foliar fungal community composition. Climate may affect the soil conditions and further alter the fungal community. Moreover, cultivation methods also will change the soil nutrients composition and the microbial network structure in the rhizosphere or foliar fungal communities. This is supported by previous studies of the cucumber rhizosphere, where AM fungi (AMF) significantly altered the nutrient composition of the branches of the host plant, with the strongest contrast observed between cucumber-irregular symbiotic plants and non-mycorrhizal cucumber plants (Ravnskov and Larsen, 2016). The composition of soil fungal communities changed with continuous cucumber cultivation, which may have been caused by the combined cultivation period of cucumber and excessive application of chemical fertilizers (Sun et al., 2021), such as nitrogen fertilizer and phosphate fertilizer.

Fungal community diversity and microbial interaction play key roles in plant growth and metabolism. Proteobacteria can directly inhibit Firmicutes from entering into the endophytic community and consequently modify the microbial community (Chen et al., 2020). Endophytes have been isolated from *Coffea canephora*, and the high biodiversity of fungal endophytes in coffee plants may help us understand the plant growth process (Vega et al., 2010). Soybean rhizosphere may act as allelochemicals in the interactions between root and soil microbial community in a long-term monocropped soybean field (Guo et al., 2011). AMF and plant growth–promoting bacteria are beneficial to horticultural crops, which could increase yield and enhance crop quality (Emmanuel and Babalola, 2020). Terpenoids are a group of structurally diverse natural products that are widely used in the flavor and fragrance industry. Furthermore, it was clarified that the fungal sesquiterpene synthase’s function differs between the phyla Ascomycota and Basidiomycota (Zhang et al., 2020).

It was evident that there is interaction between the indigenous microbial community and grain metabolism even with good-quality, mature malting barley. In the malting ecosystem, the fungal community markedly contributed to the production of microbial β-glucanases and xylanases and was also involved in proteolysis (Laitila et al., 2007). Elevated temperature also increases aphid abundance but decreases AMF colonization rates of the wheat grain, which implies that climate may affect crop quality by altering plant-associated fungal communities (Tian et al., 2019).

### Drivers in Shaping the Structure of Foliar Fungal Community

Many factors could affect the foliar fungal community structure, including plant cultivar (Martins et al., 2011), soil physical and chemical characteristics, and climate (Kauserud et al., 2013). Our results showed that planting regions with different climates and plant cultivars are key factors. The PCA results indicated that both cultivar and planting region climate could affect the community structure of foliar fungi, whereas through PERMANOVA using distance matrices (Alekseyenko, 2016), we found that the impact of ecological environment factors (*R*^2^ = 0.32, *p* < 0.05) was more significant than crop cultivar (*R*^2^ = 0.42, *p* < 0.05; Table 3).

**TABLE 3:** The impact of ecological environment factors and crop cultivar on the structure of the foliar fungal community using permutational multivariate analysis of variance using distance matrices based on Bray–Curtis similarity index.

	*df*	Sum of sequence	*R* ^2^	*F*	*p*
Planting region	1	72.83	0.32	2.61	0.0166
Cultivar	2	93.00	0.42	1.67	0.2333

The phyllosphere represents one of the most abundant habitats for microbiota colonization (Chen et al., 2020), and the role played by interactions between phyllosphere microorganisms in modifying the fungal community composition cannot be neglected. Related metastudies have identified climate as an important driving factor in different aspects of fungal biogeography, including the global distribution of common fungi and the composition and diversity of fungal communities (Vetrovsky et al., 2019). Climatic variability might modify trait selection in fungi, including spore size and dispersal characteristics. Changes in the composition and characteristics of fungal communities will have an important impact on interaction with plant communities and ecosystem functions (Andrew et al., 2016). Climate change may affect ecosystem functioning due to the narrow climatic tolerances of key fungal taxa. Mycorrhizal fungi appear to have narrower climatic tolerances than pathogenic fungi (Vetrovsky et al., 2019). Our results showed that the differences between tobacco cultivars play an important role in phyllosphere fungal community structure and affect the microbial co-occurrence pattern in phyllosphere fungal communities. Similarly, cucumber cultivars inoculated with different AMF had differential responses in terms of growth and branch nutrient composition, which revealed that plant cultivar could affect the microbial community functional diversity (Ravnskov and Larsen, 2016). Moreover, AMF also can enhance ecosystem resilience and reduce the negative impact of increased precipitation on nutrient losses (Martinez-Garcia et al., 2017). Also, mycorrhizal fungi can promote or hinder the successful spread of plants away from harsh environments (Bennett and Classen, 2020).

### Community Assembly of Foliar Fungal Community on Leave Surface

The ecological assembly process is vital for the construction of microbial communities (Sloan et al., 2006). Spatial turnover in the composition of biological communities includes (ecological) drift, selection, and dispersal. Quantitatively estimating the influences of selection, dispersal, and drift is fundamental to our understanding of ecological systems (Stegen et al., 2013). In our study, the community NRI of fungi on leaf surfaces in GC region was between 0.74 and 1.01, whereas in WZS, it was between 0.78 and 1.26; furthermore, the NTI and NRI significantly varied among cultivars and planting regions (Figure 5), which implicates that the stochastic process plays a key role in the local species diversity and spatial turnover. Other studies have previously elucidated the importance of drift in community assembly process of the legume root nodules, including the core rhizobial communities (genus *Mesorhizobium*) that were driven by dispersal limitation in concert with drift (81.1% of *nodA* communities, Ramoneda et al., 2020). During the degradation of straw, ecological drift was important across all stages of decomposition (Bao et al., 2020). The βNTI was linearly related to the sensory quality of crop leaves, indicating that the ecosystem services may depend, to some extent, on the assembly process of microbial communities. This may have resulted from stochastic processes in the assembly of foliar fungal communities. Although these results have advanced our understanding of the relationships between microbial community assembly and crop quality, future work is still required to further reveal the connections between foliar fungal community and plant molecular metabolic mechanism.

## Data Availability Statement

The original contributions presented in the study are publicly available in NCBI under accession number PRJNA778452.

## Author Contributions

ZY and JL conceived and designed the works. LX, LL, and XH conducted the experiment. LX, QZ, and HY performed the bioinformatic analyses. LX and XH performed statistical analyses. LX wrote the original draft manuscript. HY, LX, QZ, and TZ reviewed and edited the manuscript. All authors contributed to the article and approved the submitted version.

## Conflict of Interest

LX, XH, LL, HX, and TZ were employed by the China Tobacco Sichuan Industrial Co., Ltd. The remaining authors declare that the research was conducted in the absence of any commercial or financial relationships that could be construed as a potential conflict of interest.

## Publisher’s Note

All claims expressed in this article are solely those of the authors and do not necessarily represent those of their affiliated organizations, or those of the publisher, the editors and the reviewers. Any product that may be evaluated in this article, or claim that may be made by its manufacturer, is not guaranteed or endorsed by the publisher.
