# Supplementary material for: Influence of Association Network Properties and Ecological Assembly of the Foliar Fugal Community on Crop Quality
Source: Front Microbiol. 2022 Apr 5;13:783923. doi: 10.3389/fmicb.2022.783923 (PMC9037085; doi:10.3389/fmicb.2022.783923)
Supplement: Supplementary file 1 [file Data_Sheet_1.docx]

Supplementary materials for

Influence of association network properties and ecological assembly of the foliar fugal community on crop quality

This file includes:

Tables S1

Figure S1-S5

Table S1. Pearson’s correlations between net relatedness index (NRI), nearest taxon index (NTI),phylogenetic diversity (PD) and sensory quality in fungal community.

|  | NRI | | NTI | | PD | | | |
| --- | --- | --- | --- | --- | --- | --- | --- | --- |
|  | Correlation coefficient | *P* | Correlation coefficient | *P* | | Correlation coefficient | *P* |  |
| Fragrance: bean | -0.188 | 0.455 | -0.043 | 0.864 | | -0.287 | 0.249 |  |
| Fragrance: woody | 0.186 | 0.461 | -0.120 | 0.635 | | -0.318 | 0.198 |  |
| Fragrance: sweet | -0.297 | 0.232 | 0.450 | 0.061 | | -0.263 | 0.292 |  |
| Fragrance: burnt sweet | **0.525** | **0.025** | 0.130 | 0.607 | | -0.317 | 0.199 |  |
| Fragrance: honey | 0.065 | 0.798 | -0.331 | 0.180 | | 0.419 | 0.083 |  |
| Fragrance: normal | -0.123 | 0.628 | -0.108 | 0.671 | | -0.012 | 0.962 |  |
| Fragrance: floral | -0.368 | 0.133 | 0.256 | 0.305 | | -0.082 | 0.747 |  |
| Fragrance: milk | **-0.541** | **0.020** | 0.024 | 0.926 | | -0.190 | 0.451 |  |
| Fragrance: resin | 0.102 | 0.686 | -0.105 | 0.678 | | **0.663** | **0.003** |  |
| Fragrance: cellar | -0.105 | 0.678 | **0.509** | **0.031** | | -0.300 | 0.227 |  |
| Fragrance: baking | -0.418 | 0.085 | -0.198 | 0.432 | | -0.139 | 0.582 |  |
| Fragrance: hay | 0.392 | 0.108 | -0.119 | 0.639 | | -0.428 | 0.077 |  |
| Fragrance: leather | -0.062 | 0.808 | 0.445 | 0.065 | | -0.073 | 0.774 |  |
| Fragrance: rouge | 0.107 | 0.674 | -0.214 | 0.394 | | **0.907** | **0.000** |  |
| Miscellaneous gas: protein smell | 0.167 | 0.508 | -0.112 | 0.657 | | -0.328 | 0.183 |  |
| Miscellaneous gas: soil fishy gas | **0.598** | **0.009** | 0.177 | 0.483 | | -0.316 | 0.201 |  |
| Miscellaneous gas: green mixed gas | -0.115 | 0.648 | -0.318 | 0.198 | | -0.487 | 0.040 |  |
| Miscellaneous gas: burnt gas | 0.314 | 0.204 | 0.074 | 0.769 | | **-0.501** | **0.034** |  |
| Miscellaneous gas: woody gas | -0.399 | 0.101 | -0.285 | 0.252 | | -0.054 | 0.830 |  |
| Mellowness of aroma: mellowness | 0.045 | 0.858 | 0.362 | 0.140 | | 0.280 | 0.260 |  |
| Mellowness of aroma: richness | 0.162 | 0.522 | 0.436 | 0.071 | | 0.043 | 0.867 |  |
| Mellowness of aroma: maturity | 0.071 | 0.778 | 0.385 | 0.114 | | 0.119 | 0.638 |  |
| Mellowness of smoke: fullness | -0.030 | 0.905 | 0.372 | 0.128 | | 0.228 | 0.363 |  |
| Mellowness of smoke: smoothness | -0.030 | 0.905 | 0.372 | 0.128 | | 0.228 | 0.363 |  |
| Mellowness of smoke: lingering | -0.014 | 0.956 | 0.320 | 0.196 | | 0.356 | 0.147 |  |
| Mellow aftertaste: sweetness | 0.044 | 0.864 | 0.315 | 0.203 | | 0.428 | 0.076 |  |
| Mellow aftertaste: irritation | 0.314 | 0.204 | 0.266 | 0.285 | | 0.465 | 0.052 |  |
| Mellow aftertaste: cleanliness | 0.071 | 0.780 | 0.273 | 0.273 | | 0.408 | 0.093 |  |
| Mellow aftertaste: aftertaste | -0.198 | 0.432 | 0.377 | 0.123 | | -0.059 | 0.817 |  |
| Combustion characteristics: combustibility | 0.223 | 0.374 | 0.131 | 0.604 | | **0.514** | **0.029** |  |
| Combustion characteristics: gray | 0.208 | 0.408 | 0.059 | 0.815 | | 0.300 | 0.226 |  |
| Combustion characteristics: condensed gray | 0.377 | 0.123 | 0.200 | 0.426 | | 0.164 | 0.516 |  |
| Coordination of flammability indicators: sense of balance | 0.241 | 0.335 | 0.193 | 0.444 | | 0.306 | 0.216 |  |


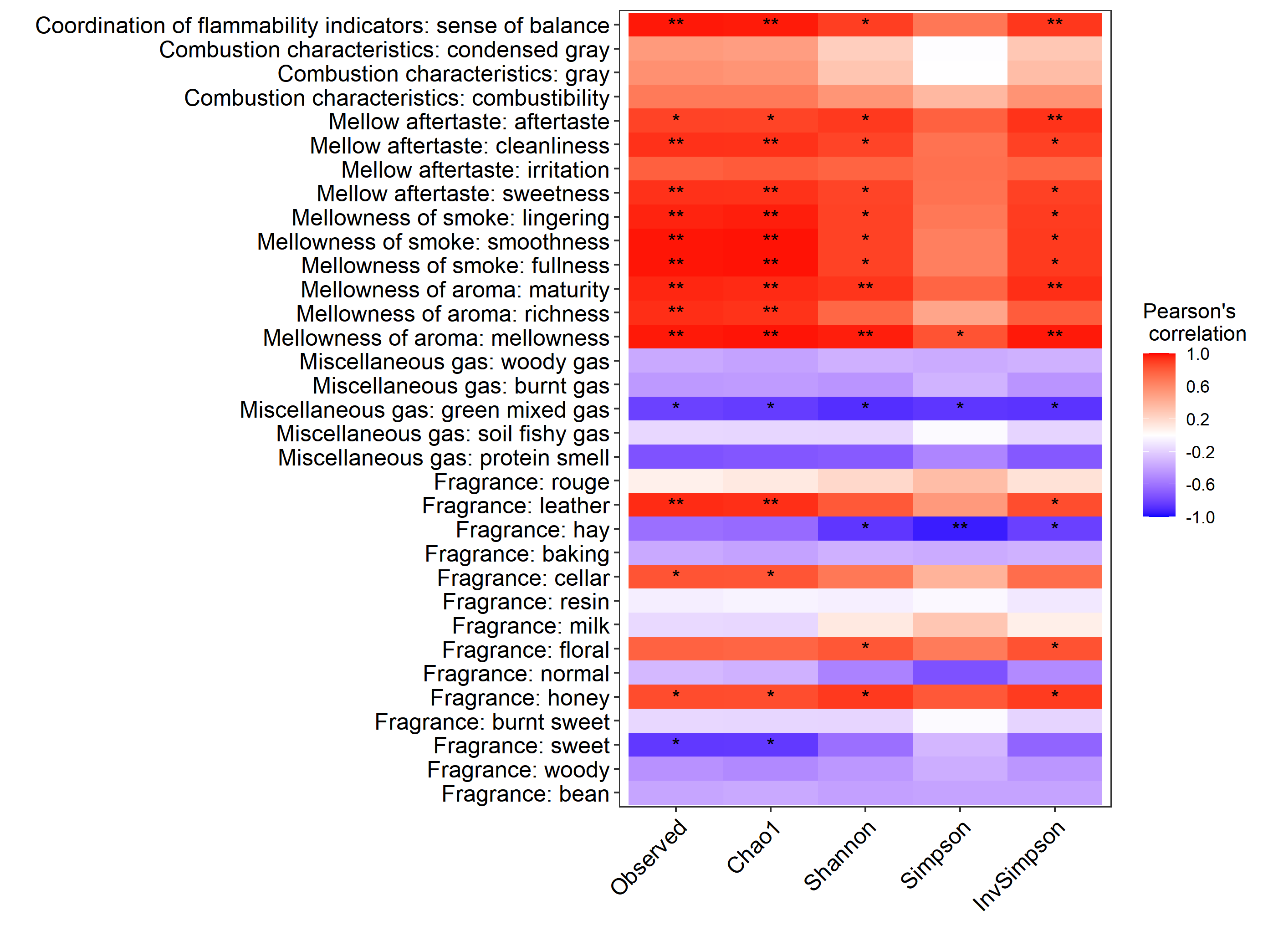


Figure S1. Pearson’s correlations between foliar fungi diversity and sensory quality. Color denotes the strength of Pearson’s correlations. *: P< 0.05; **: P< 0.01.


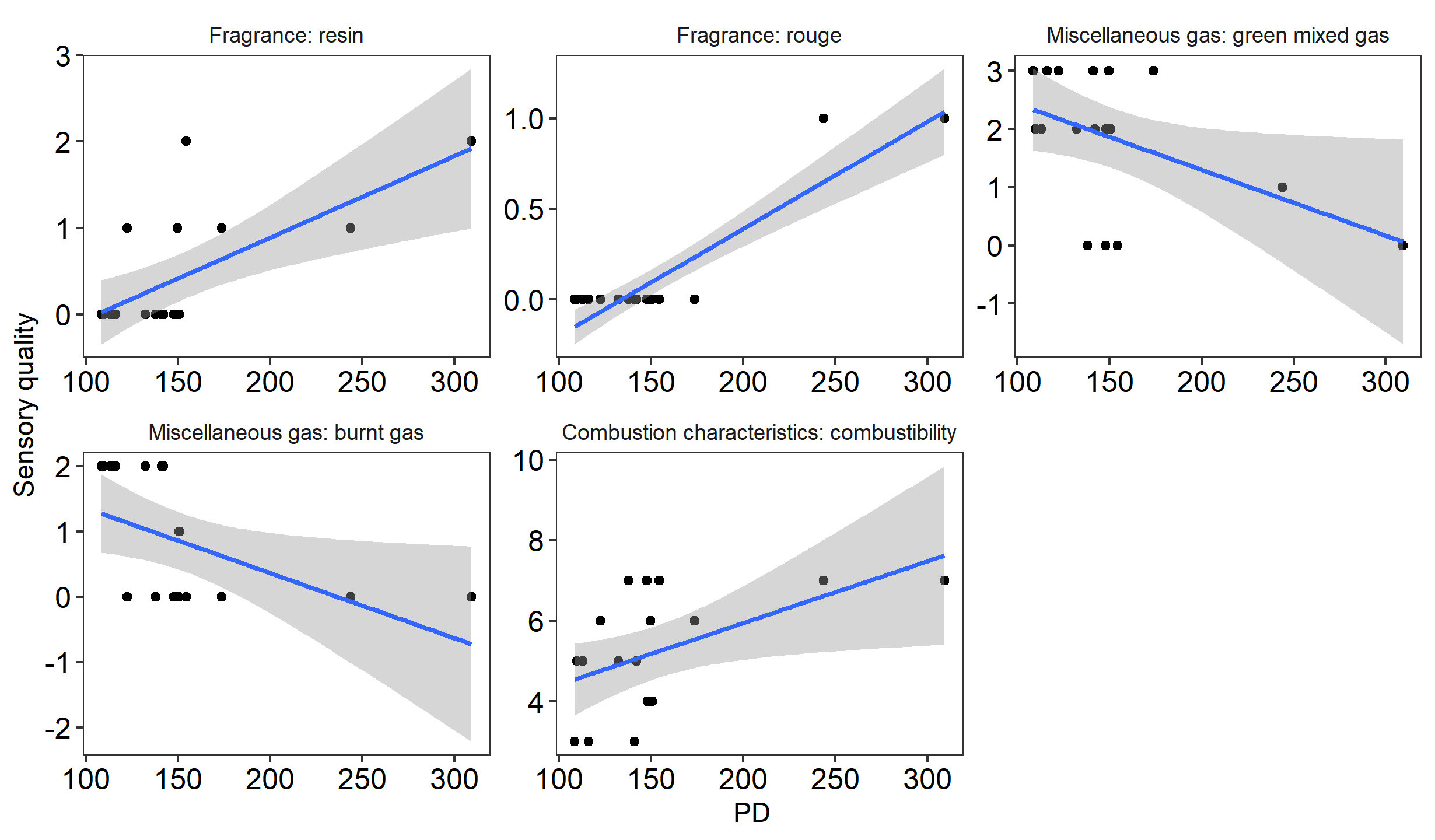


Figure S2. Linear regression analyses for the relationships between phylogenetic diversity (PD) and sensory quality.


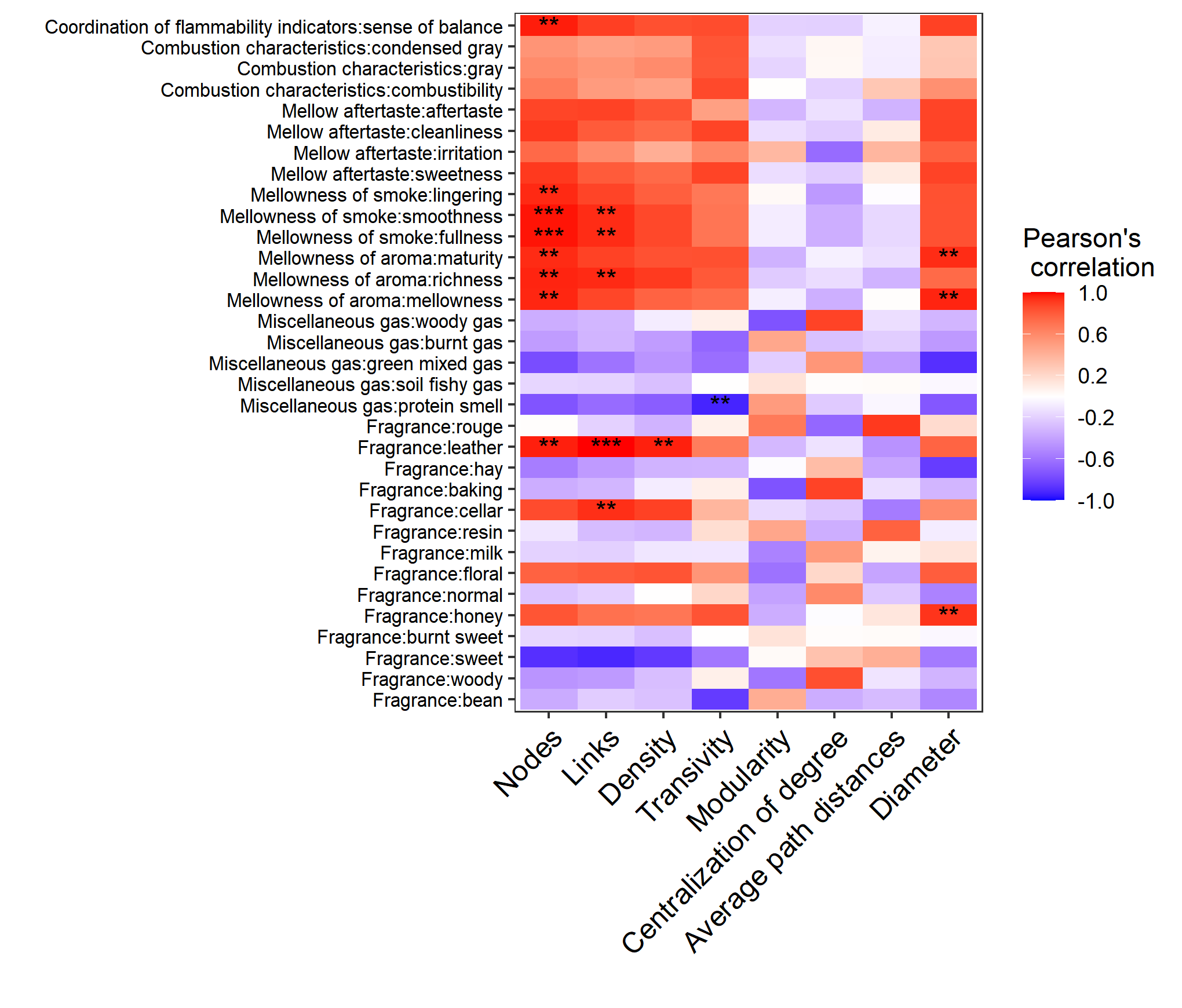


Figure S3. Heatmap for Pearson’s correlations between network properties and sensory quality. Colors denotes the strength of Pearson’s correlations.


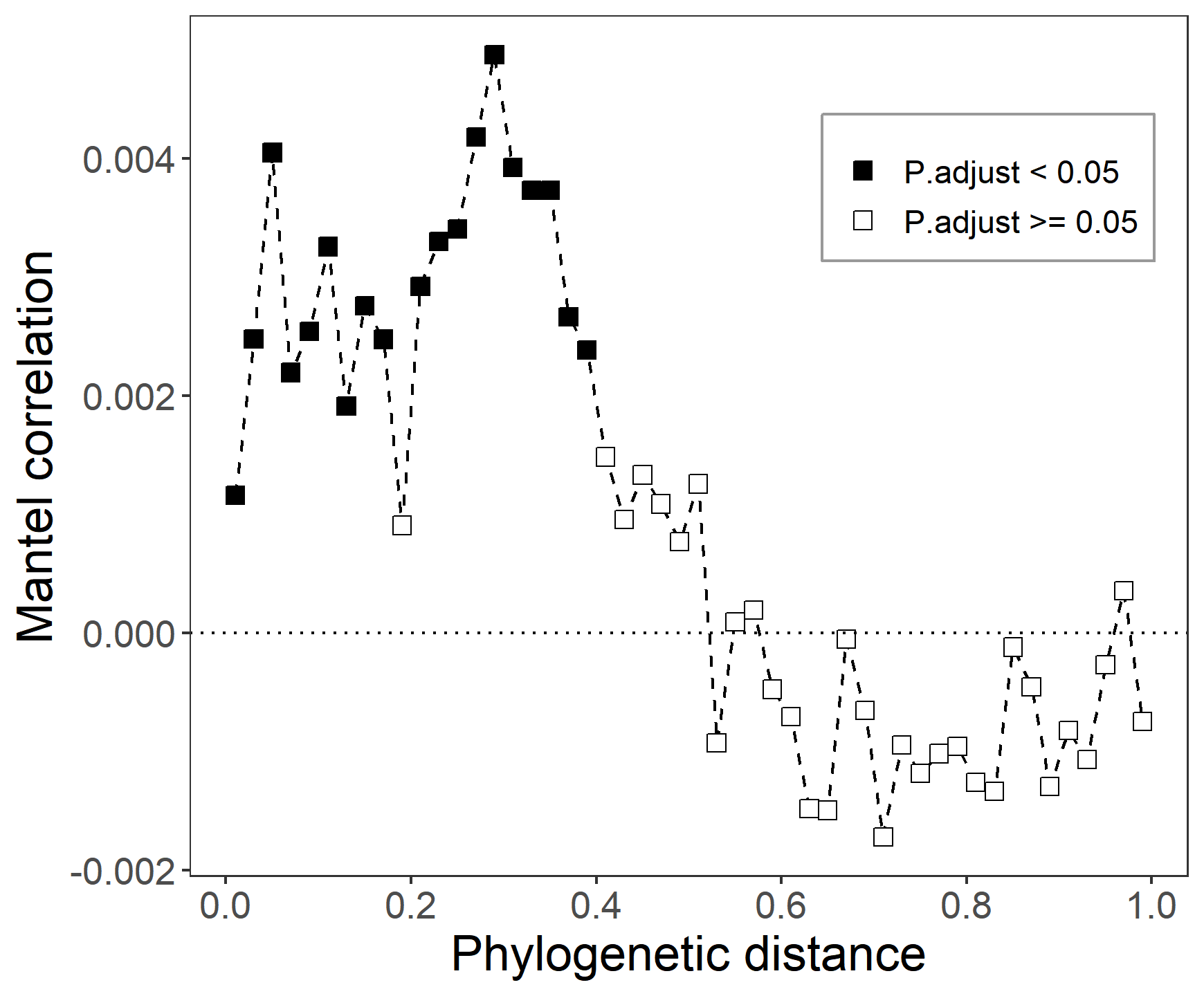


Figure S4. Phylogenetic signal test for foliar fungi communities. The filled squares denoted the mantel correlation between environmental factors and phylogenetic structure are significant (P < 0.05).

D
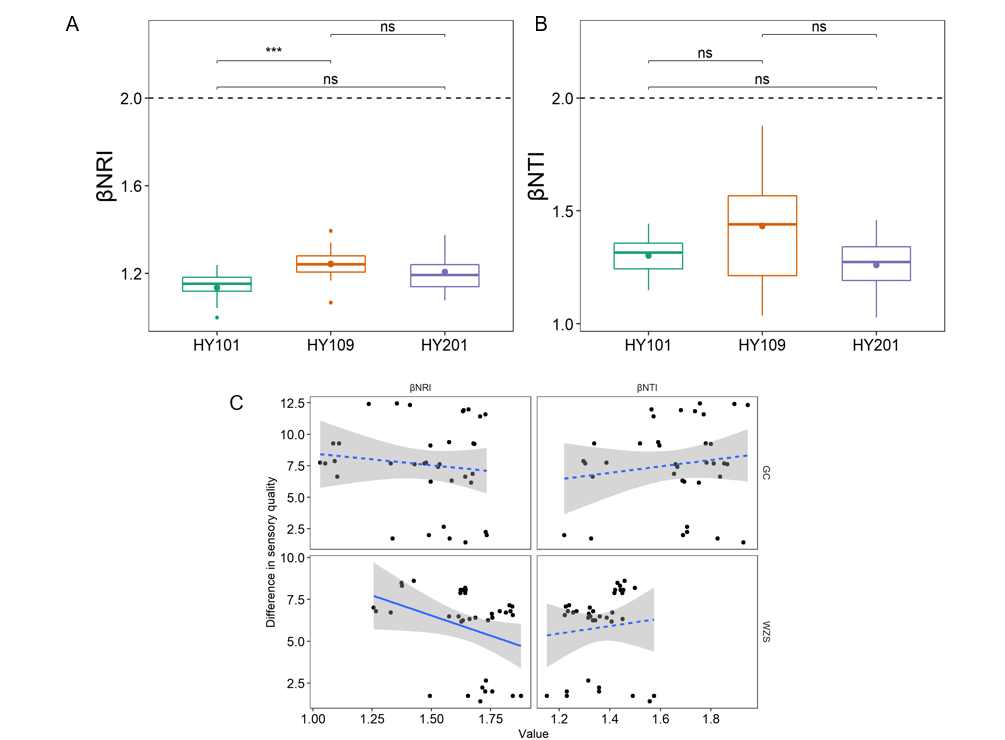


Figure S5. (A) Beta net relatedness index(βNRI).(B) beta nearest taxon index (βNTI) of foliar fungi communities on crop leaves across different crop cultivars. (C) Linear regression analyses for the relationships between βNRI or βNTI and differences in sensory quality of crop leaves across the two regions. *** :P<0.001.
